# Developing and comparing deep learning and machine learning algorithms for osteoporosis risk prediction

**DOI:** 10.3389/frai.2024.1355287

**Published:** 2024-06-11

**Authors:** Chuan Qiu, Kuanjui Su, Zhe Luo, Qing Tian, Lanjuan Zhao, Li Wu, Hongwen Deng, Hui Shen

**Affiliations:** Tulane Center for Biomedical Informatics and Genomics, Deming Department of Medicine, School of Medicine, Tulane University, New Orleans, LA, United States

**Keywords:** osteoporosis, bone mineral density, machine learning, deep learning, disease prediction

## Abstract

**Introduction:**

Osteoporosis, characterized by low bone mineral density (BMD), is an increasingly serious public health issue. So far, several traditional regression models and machine learning (ML) algorithms have been proposed for predicting osteoporosis risk. However, these models have shown relatively low accuracy in clinical implementation. Recently proposed deep learning (DL) approaches, such as deep neural network (DNN), which can discover knowledge from complex hidden interactions, offer a new opportunity to improve predictive performance. In this study, we aimed to assess whether DNN can achieve a better performance in osteoporosis risk prediction.

**Methods:**

By utilizing hip BMD and extensive demographic and routine clinical data of 8,134 subjects with age more than 40 from the Louisiana Osteoporosis Study (LOS), we developed and constructed a novel DNN framework for predicting osteoporosis risk and compared its performance in osteoporosis risk prediction with four conventional ML models, namely random forest (RF), artificial neural network (ANN), k-nearest neighbor (KNN), and support vector machine (SVM), as well as a traditional regression model termed osteoporosis self-assessment tool (OST). Model performance was assessed by area under ‘receiver operating curve’ (AUC) and accuracy.

**Results:**

By using 16 discriminative variables, we observed that the DNN approach achieved the best predictive performance (AUC = 0.848) in classifying osteoporosis (hip BMD T-score ≤ −1.0) and non-osteoporosis risk (hip BMD T-score > −1.0) subjects, compared to the other approaches. Feature importance analysis showed that the top 10 most important variables identified by the DNN model were weight, age, gender, grip strength, height, beer drinking, diastolic pressure, alcohol drinking, smoke years, and economic level. Furthermore, we performed subsampling analysis to assess the effects of varying number of sample size and variables on the predictive performance of these tested models. Notably, we observed that the DNN model performed equally well (AUC = 0.846) even by utilizing only the top 10 most important variables for osteoporosis risk prediction. Meanwhile, the DNN model can still achieve a high predictive performance (AUC = 0.826) when sample size was reduced to 50% of the original dataset.

**Conclusion:**

In conclusion, we developed a novel DNN model which was considered to be an effective algorithm for early diagnosis and intervention of osteoporosis in the aging population.

## Introduction

Osteoporosis is a systemic skeletal disorder characterized by low bone mineral density (BMD) and microarchitectural deterioration of bone tissue, resulting in an increased risk of bone fragility and susceptibility to fracture (Kanis, 2002). It has become an increasingly serious public health concern, especially in the aging population (Poole and Compston, 2006). In the United States, it is estimated that over 10.2 million older people have suffered from osteoporosis (Wright et al., 2014). Osteoporosis-related fractures, particularly hip fractures, are one of the leading causes of disability and mortality worldwide and resulted in an enormous social and economic burden on the society (Johnell and Kanis, 2004). Early diagnosis of osteoporosis risk is challenging. In the past two decades, several traditional epidemiological studies have identified a range of risk factors for osteoporosis and its related fractures, the most well-known factors include older age, low body weight, history of fracture, estrogen deficiency at an early age, low calcium intake, and vitamin D deficiency (Khosla and Melton, 2007). At present, dual-energy X-ray absorptiometry (DXA) is generally deemed as the gold standard tool for diagnosing osteoporosis. However, mass screening of subjects with high osteoporosis risk in the general population using DXA is not widely recommended because of the relatively high cost of the DXA scan. In addition, the availability of DXA scanner is relatively limited in most rural areas. Recently, the introduction of Osseus, a prototype device that measures BMD using non-ionizing microwave electromagnetic radiation, aims to overcome the cost and accessibility challenges associated with DXA (Albuquerque et al., 2022). Nevertheless, further validation against established gold standard methods is needed to confirm Osseus’ effectiveness in early osteoporosis detection. To surmount this hurdle, a couple of epidemiological studies have attempted to develop algorithms/tools for predicting osteoporosis risk using demographic and routinely collected clinical data (Koh et al., 2001). For example, the most widely used tool, namely the osteoporosis self-assessment tool (OST), relies on a simple regression model with age and body weight to predict osteoporosis risk (Koh et al., 2001). Nevertheless, this tool has a relatively low accuracy in clinical implementation (Raisz, 2005), resulting in a large number of subjects who were at high risk of osteoporosis failed to be identified, while other subjects with relatively low osteoporosis risk were given unnecessary medical interventions. Although several more complex models have been proposed later by incorporating some other factors, such as family history, physical activity, and occupational risk etc., these models have not shown significantly improved performance in predicting osteoporosis risk compared to OST (Rud et al., 2009). Meanwhile, predicting dataset with massive sample size and high dimensionality may introduce several challenges, such as overfitting, heterogeneity, noise accumulation, and spurious correlation, which make conventional statistical methods inappropriate and unreliable in model development (Fan et al., 2014). Therefore, the state-of-the-art algorithms that can better discriminate osteoporosis risk and determine more nuanced relationships between risk factors and outcome need to be explored.

Machine learning (ML) is an area of artificial intelligence which can use a set of advanced algorithms for data classification without stringent statistical assumptions (Kotsiantis et al., 2006; Jordan and Mitchell, 2015). It offers a powerful alternative approach to conventional prediction modeling. ML relies on a computer to learn all complex and non-linear interactions which is more probable scenario for numerous biological systems (Loscalzo et al., 2007; Sturmberg et al., 2012; Turnbull et al., 2018; Li et al., 2019; Medina-Ortiz et al., 2020; Manicka et al., 2023) between variables by minimizing the error between predicted and observed outcomes (Dreiseitl and Ohno-Machado, 2002). In addition to potentially improving prediction, ML may identify latent variables, which are unlikely to be observed but might be inferred from other observable variables. In the past decade, a branch of ML algorithms (Table 1), such as random forest (RF), artificial neural network (ANN), k-nearest neighbor (KNN), and support vector machine (SVM) etc., have been widely applied in clinical medicine and have shown higher accuracy for diagnosis than conventional approaches (Hsieh et al., 2011). In the bone-related field, several studies have indicated that supervised ML can help forecast low BMD or fractures (Sadatsafavi et al., 2005; Eller-Vainicher et al., 2011; Xu et al., 2013). For instance, it was reported that the performance of an ANN model for BMD prediction in postmenopausal women was superior to the conventional regression methods (Sadatsafavi et al., 2005). Also, ANN showed a better performance for predicting morphometric vertebral fractures in postmenopausal osteoporosis than logistic regression analysis (Eller-Vainicher et al., 2011). Furthermore, Xu et al. (2013) suggested that a SVM model of several combined features can be a useful tool for the early diagnosis and intervention for women with osteoporosis.

**Table 1:** Supervised ML algorithms for disease prediction and/or classification.

Approach	Type of supervised learning	Description	Graphical depiction
RF ^#^	Classification or regression	RF builds multiple decision trees and merges them together to get a more accurate prediction. The output is the mode of the predicted class (classification) or the mean of the predicted class (regression) of the individual trees	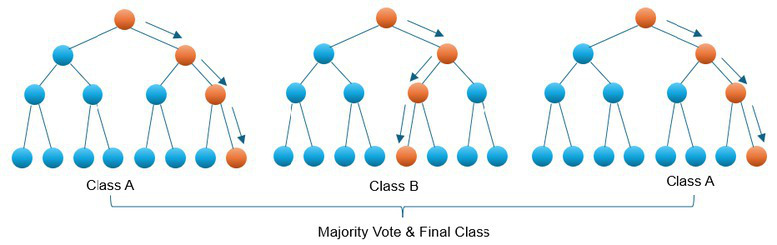
ANN	Classification	An ANN consists of units (neurons) arranged in layers, with the aim of converting an input vector into some output. The layers between the input and output layers are often hidden. Each unit takes an input, applies a (often nonlinear) function to it and passes it onto the next layer. Weights are applied to the signals passing from one unit to another, which are modified during the training phase	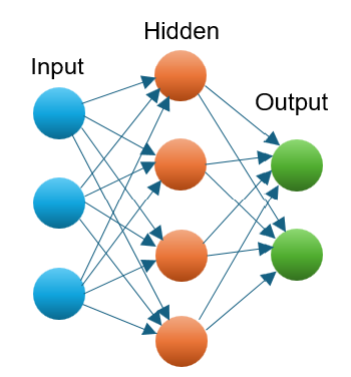
KNN	Classification or regression	The KNN algorithm is a nonparametric method (that is, it makes no assumptions on the underlying data distribution). The algorithm is based on feature similarity (that is, how closely a new item resembles each item in the training set). The item is classified by a majority vote of its neighbors (that is, the new item is assigned to the class most common among its neighbors)	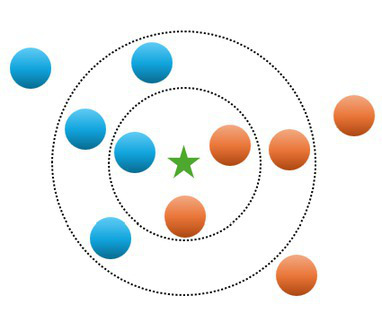
SVM	Classification	The goal of a SVM is to identify a hyperplane that best divides the data into the classes This hyperplane could be a line (for separating 2D data), a plane (for separating 3D data) or a hyperplane (for separating 4D data) The support vector machine finds the coefficients that result in the best separation of the classes by trying to maximize the margin between the hyperplane and the closest points to the hyperplane	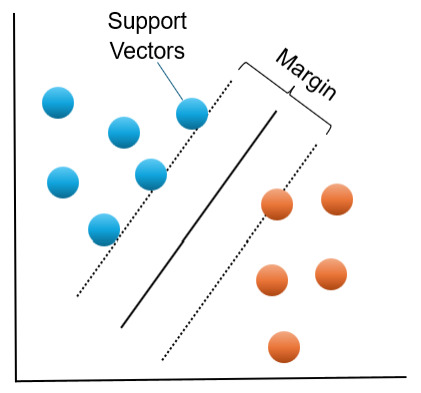

ML, machine learning; RF, Random forest; ANN, artificial neural network; KNN, k-nearest neighbor; SVM, support vector machine.

# Can be used for feature selection.

More recently, a new class of ML methods, namely deep learning (DL) or deep neural network (DNN), has gained much attention and achieved impressive and sometimes, breakthrough, results across a variety of artificial intelligence tasks (LeCun et al., 2015). DL is inspired by the ability of human brain to abstract high-level representations from low-level sensory stimuli; these multi-leveled representations can be casted mathematically as multi-layered neural networks, it is being able to be trained via layer-wise back-propagation to obtain tractable optimization (LeCun et al., 2015). DL is currently the state-of-the-art method in the areas of image recognition (Krizhevsky et al., 2012; Tompson et al., 2014) and speech recognition (Hinton et al., 2012). It has also produced promising results in reconstructing brain circuits (Helmstaedter et al., 2013) and natural language understanding (Collobert et al., 2011). Interestingly, DL has also gained tremendous successes in various areas of genomics research (Angermueller et al., 2016; Min et al., 2017), such as predicting the intrinsic molecular subtypes of breast cancer (Tan et al., 2015), inferring expression profiles of genes (Chen et al., 2016), and predicting the functional activity of genomic sequence (Kelley et al., 2016). Furthermore, DL algorithms have been adopted to disease risk prediction and/or classification for several common diseases, such as cardiovascular disease (Poplin et al., 2018) and eye disease (Grassmann et al., 2018). However, few DL algorithms were applied to osteoporosis risk prediction, and it is still unclear whether such techniques can outperform other conventional ML algorithms in bone study. Therefore, it is intuitively appealing to systematically investigate and compare the performance of DL and other ML algorithms in prediction of osteoporosis risk, especially in large-scale populations.

In this study, by leveraging extensive BMD and other demographic and clinical data from our Louisiana Osteoporosis Study (LOS) (du et al., 2017), we developed and constructed a novel DNN framework for predicting osteoporosis risk and compared its performance with four other commonly used ML models (i.e., RF, ANN, KNN, and SVM), as well as the traditional regression model OST. We demonstrated that the DNN model can more accurately predict the osteoporosis risk in the aging population, which may facilitate and increase the effectiveness of early diagnosis and prevention of this disease.

## Materials and methods

### Subjects

All the subjects used in this study were recruited through LOS (du et al., 2017), a repertoire of more than 17,000 subjects (by end of October 2023) collected for investigating genetic and environment risk and protective factors for osteoporosis in Southern Louisiana. Subjects aged 18 and over were recruited in New Orleans, Baton Rouge, and surrounding areas in Louisiana, USA. An extensive set of exclusion criteria (Supplementary materials) was adopted for the LOS recruitment to exclude subjects with known diseases/conditions that may affect bone metabolism. In the current study, we focused on 9,185 subjects, aged 40 years or older, who are at risk of developing osteoporosis as bone density gradually decreases due to changes in the bone remodeling process. We removed subjects of race/ethnicity with small sample size (372 subjects of Native American/Pacific Islander and other) to mitigate the potential influences on the prediction performance and generalization of ML models, and participants who have inadequate numbers of available clinical measurements (679 subjects with more than 4 missing variables). Ultimately, a total of 8,134 subjects were encompassed in this study, including 3,541 males and 4,593 females from Caucasian/White, African-American/Black, Asian, and Hispanic/Latino. The detailed characteristics of the exquisitely selected study subjects were summarized in Table 2. This study was approved by the Institutional Review Boards for Human Investigation at Tulane University (New Orleans, USA), and the signed informed-consent documents were obtained from all study participants before any data Research Topic.

**Table 2:** The basic characteristics of the study subjects with 16 most discriminative predicting features.

Variables	Total (*n* = 8,134)	Osteoporosis-risk Group (*n* = 2,004)	Non-osteoporosis risk group (*n* = 6,130)
Age (year)	55.12 (9.77)	59.95 (10.75)	53.54 (8.87)
Height (cm)	167.31(9.38)	164.66 (9.19)	168.18 (9.27)
Weight (kg)	79.04 (19.54)	66.01 (13.18)	83.30 (19.39)
Diastolic_Pressure (mmHg)	79.28 (12.25)	76.79 (12.15)	80.01 (12.17)
Heart_Rate (times/min)	72.95 (11.91)	72.77 (11.56)	73.01 (12.02)
Grip_Strength (kg)	31.38 (12.41)	27.15 (10.62)	32.76 (12.64)
Num_Exercises_Weekly (times/week)	2.96 (2.70)	3.07 (2.76)	2.92 (2.67)
Smoke_Year (year)	14.50 (16.79)	16.10 (18.18)	13.79 (12.28)
Sex (male/female)	3,541/4,593	752/1,252	2,789/3,341
Race/Ethnicity ^#^	1 (*n* = 4,517), 2 (*n* = 2,887), 3 (*n* = 517), 4 (*n* = 213)	1 (*n* = 752), 2 (*n* = 1,252), 3 (*n* = 130), 4 (*n* = 50)	1 (*n* = 3,212), 2 (*n* = 2,368), 3 (*n* = 387), 4 (*n* = 163)
Sun_Exposure (no/yes)	1,524/6,610	423/1,581	1,101/5,029
Alcohol_Drink (no/yes)	2,530/5,604	652/1,352	1,878/4,252
Beer_Drink (no/yes)	4,725/3,409	1,269/735	3,456/2,764
Fracture_History (no/yes)	5,779/2,355	746/1,258	4,521/1,609
Economic_Level ^#^	1 (*n* = 2,201), 2 (*n* = 912), 3 (*n* = 740), 4 (*n* = 454), 5 (*n* = 906), NA (*n* = 2,921)	1 (*n* = 520), 2 (*n* = 231), 3 (*n* = 158), 4 (*n =* 106), 5 (*n* = 227), NA (*n* = 762)	1 (*n* = 1,681), 2 (*n* = 681), 3 (*n* = 582),4 (*n* = 348), 5 (*n* = 679), NA (*n* = 2,159)
Education_Level ^#^	1 (*n* = 230), 2 (*n* = 1,962), 3 (*n* = 2,775), 4 (*n* = 1,043), NA (*n* = 2,124)	1 (*n* = 54), 2 (*n* = 472), 3 (*n* = 671), 4 (*n* = 277), NA (*n* = 530)	1 (*n* = 176), 2 (*n* = 1,594), 3 (*n* = 2,104), 4 (*n* = 766), NA (*n* = 1,696)

Continuous variables were presented as mean (SD).

# The race was categorized into Caucasian/White, African-American/Black, Asian, and Hispanic/Latino, which were coded as 1, 2, 3, and 4, respectively. The economic levels (personal annual income) were categorized into under $20,000, $20,000–39,999, $40,000–59,999, $60,000 − 79,999, and $80,000 or more, which were coded as 1, 2, 3, 4, and 5, respectively. The education levels were categorized into less than high school graduate, high school graduate, college (including some college and college graduate), and graduate level, which were coded as 1, 2, 3, and 4, respectively. NA indicates the missing data.

### Measurements

A total of 23 potential risk factors including demographic and anthropometric measurements, lifestyle factors, and medical history were assessed by questionnaires for all subjects. The detailed measurement and labeling process for each potential risk factor was described in Supplementary materials. In this study, we focused on total hip BMD, as low hip BMD is a major risk factor of hip fractures which are one of the leading causes of disability and mortality worldwide and resulted in an enormous social and economic burden on the society (Johnell and Kanis, 2004). The BMD was measured with Hologic Discover-A DXA machine (Hologic Inc., Bedford, MA, United States) by trained and certified research staff. The machine was calibrated daily, and software and hardware were kept up to date during the data Research Topic process. The measurement precision, as reflected by coefficients of variation for total hip BMD, was approximately 1.0%. More details on data quality control including the usual covariation for repeated measures have been described in our earlier publication (Deng et al., 1999). The osteoporosis risk of each subject was defined based on his/her hip BMD *T*-score value, which is expressed as the number of standard deviations of a person’s measured BMD above or below the mean BMD value of young adults of the same sex and ethnicity (Watts, 2004). We categorized the subjects into an osteoporosis risk group (hip BMD T-score ≤ −1.0) and a non-osteoporosis risk group (hip BMD T-score > −1.0).

### Data preprocessing

The dataset was preprocessed to improve the performance of model prediction. Initially, all the missing data and outliers were imputed using predictive mean matching algorithm (Morris et al., 2014) through R package *mice*.^1^ Due to the high dimensionality of the input variables, we calculated and visualized the Pearson’s correlations between all 14 continuous variables through R package *corrplot*^2^ and removed variables with correlation coefficient larger than 0.70. In addition, we performed a robust feature selection analysis on the remaining variables based on genetic algorithm (Muni et al., 2006) to determine the best subset of relevant variables for model construction. Genetic algorithm is relatively insensitive to noise, and can not only improve the model classification performance but also increase the model interpretability (Saeys et al., 2007). Finally, the most discriminative variables identified by genetic algorithm were selected for final model building.

### DL algorithm

DL refers to DNN framework, which is widely applied in pattern recognition, image processing, computer vision, and recently in bioinformatics (Min et al., 2017). Similar to other feed-forward artificial neural networks, DL employs more than one hidden layer (*y*) that connects the input (*x*) and output layer (*z*) via a weight (*W*) matrix as shown in Eq. (1). Here we used sigmoid function as the activation function:


(1) $$y=\mathrm{sigmoid}\left(Wx+b\right)$$

Activation value of the hidden layer (*y*) can be calculated by sigmoid of the multiplication of the input sample *x* with the weight matrix *W* and bias *b*. The transpose of the weight matrix *W* and the bias *b* can then be used to construct the output (*z*) layer, as described in Eq. (2):


(2) $$z=\mathrm{sigmoid}\left(W^{\prime }y+b^{\prime }\right)$$

The best set of the weight matrix *W* and bias *b* is expected to minimize the difference between the input layer (*x*) and the output layer (*z*). The objective function is called cross-entropy in Eq. (3) below, in which the optimal parameters are obtained by stochastic gradient descent searching:


(3) $$L_{H}\left(x,z\right)=-\overset{d}{\underset{k=1}{\sum }}\left[x_{k}\log\;z_{k}+\left(1-x_{k}\right)\log\left(1-z_{k}\right)\right]$$

To train the model, we first supplied sample input (*x*) to the first layer and obtained the best parameters (*W*, *b*) and the activation of the first hidden layer (*y*), and then used *y* to learn the second layer. We repeated this process in subsequent layers, updating the weights and bias in each epoch. We then used back-propagation to tune the parameters of all layers. Finally, we fed the output of the last hidden layer to a softmax classifier which assigned new labels to the samples.

### Modeling and evaluation

We randomly split the dataset into 80% training set and 20% testing set. The 80/20 split is a common practice of splitting ratio for samples of moderate size in the ML applications. To avoid sampling bias, we performed 10-fold cross-validation scheme on the 80% training set during the model construction process and tested the model on the hold out 20% of data. In the realm of binary classification tasks, the classification threshold represents the pivotal probability threshold utilized to differentiate between positive (osteoporosis risk) and negative (non-osteoporosis risk) classifications for observations. In our classification testing, we adhered to the default threshold of 0.5. Specifically, this threshold dictates that if the predicted probability of osteoporosis risk surpasses or equals 0.5, the observation is classified as positive (osteoporosis risk); conversely, if the predicted probability falls below 0.5, it is classified as negative (non-osteoporosis risk). The overall performance of the models was assessed by area under a receiver-operating characteristic (ROC) curve (AUC). We repeated the splitting process ten times and calculated the average AUC on the 10 hold out testing sets. The performance of the ML algorithms was also evaluated by accuracy, sensitivity, and specificity. The sensitivity or true positive rate (TPR) is defined as the percentage of participants who are correctly identified as having the disease, as described in Eq. (4):


(4) $$\mathrm{Sensitivity}=\frac{\mathrm{True}\;\mathrm{Positive}}{\mathrm{True}\;\mathrm{Positive}+\mathrm{False}\;\mathrm{Negative}}$$

The specificity or true negative rate (TNR) is defined as the percentage of participants who are correctly identified as being healthy, as described in Eq. (5):


(5) $$\mathrm{Specificity}=\frac{\mathrm{True}\;\mathrm{Negative}}{True\;\mathrm{Negative}+\mathrm{False}\;\mathrm{Positive}}$$

The quantity 1-specificity is the false positive rate and is the percentage of participants that are incorrectly identified as having the disease. The accuracy is defined as the percentage of participants who are correctly identified as being healthy and having the disease, as described in Eq. (6):


(6) $$\mathrm{Accuracy}=\frac{\mathrm{True}\;\mathrm{Positive}+\mathrm{True}\;\mathrm{Negative}}{\begin{array}{l} \mathrm{True}\;\mathrm{Positive}+\mathrm{True}\;\mathrm{Negative}+\\ \mathrm{False}\;\mathrm{Positive}+\mathrm{False}\;\mathrm{Negative} \end{array}}$$

To control overfitting, we tuned DNN model on the following regularization parameters: Epochs (number of passes of the full training set), *l*1 (increases model stability, penalty to converge many weights to 0), *l*2 (penalty to prevent weights enlargement), input dropout ratio (ratio of ignored neurons in the input layer during training), and number of hidden layers. We used R package *h2o* (www.h2o.ai/) to develop the novel DL model and tune the parameters. For comparisons, we selected OST model and a representative set of ML algorithms that are commonly used for risk prediction by the clinical community, including RF, ANN, KNN, and SVM. The model development and parameter tuning for OST and the selected four ML algorithms were carried out through R package *caret.*^3^

Datasets used in this study were class-imbalanced because the non-osteoporosis risk group contained significantly more samples than the osteoporosis risk group. Applying a classifier to the imbalanced data can result in erroneous prediction (heavily biased toward the majority class). To obtain the optimal result, we adopted a sampling-based approach for rebalancing the data (He and Garcia, 2009; Haixiang et al., 2017).

### Feature importance analysis

To test the relative contribution of each factor to osteoporosis risk, we performed feature importance analysis through the embedded method of each ML algorithm. In these approaches, features that provided unique information to the trained model were ranked more important than those giving redundant information. The variable importance functions “varimp” in *h2o* and “varImp” in *caret* R packages were used to rank features for the developed novel DNN and all other models, respectively.

### Subsampling experiments

To understand the impact of training information on model prediction, we further performed two additional subsampling experiments: (1) randomly removing 25, 50, and 75% of the data sets to assess the effect of sample size on model prediction; (2) using only the top 2, 5, and 10 most important variables to identify the most effective risk factors for model prediction. The new model was trained for each scenario and its performance was evaluated on a separate testing set with 10-fold cross-validation scheme.

## Results

### The predictive performance of the developed novel DNN model outperforms other conventional ML algorithms

A total of 8,134 subjects (*n* = 3,541 males and 4,593 females), aged 40 years or older, were retrieved from LOS data set, of which 2,004 (24.6%) subjects had high osteoporosis risk (hip BMD T-score ≤ −1). To smooth out the noise and simplify the model, we first dropped out 3 continuous variables, including body mass index (BMI), waist circumference, and hip circumference, which were highly correlated with weight (Pearson’s correlations >0.70) (Supplementary Figure 1). To detect the influence of the remaining 20 variables on the BMD variation, we implemented feature selection analysis by genetic algorithms and further removed 4 potential risk factors including milk drinking, cheese intake, systolic pressure, and wine drinking. Finally, 16 most discriminative variables (Table 2, Supplementary Figure 2) were selected for model building.

**Figure 1:**
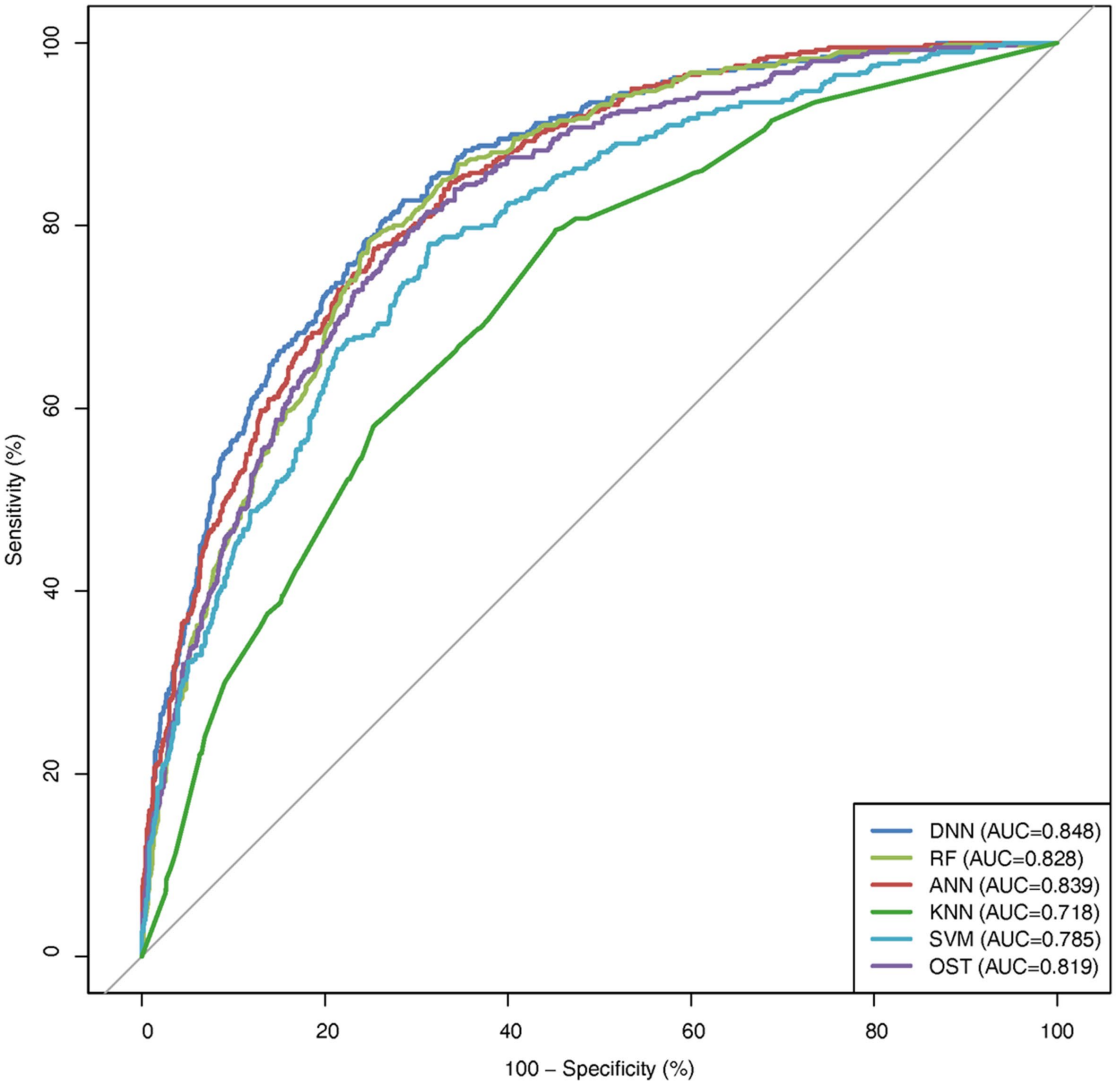
ROC curve comparison among DNN, OST, and other ML models.

**Figure 2:**
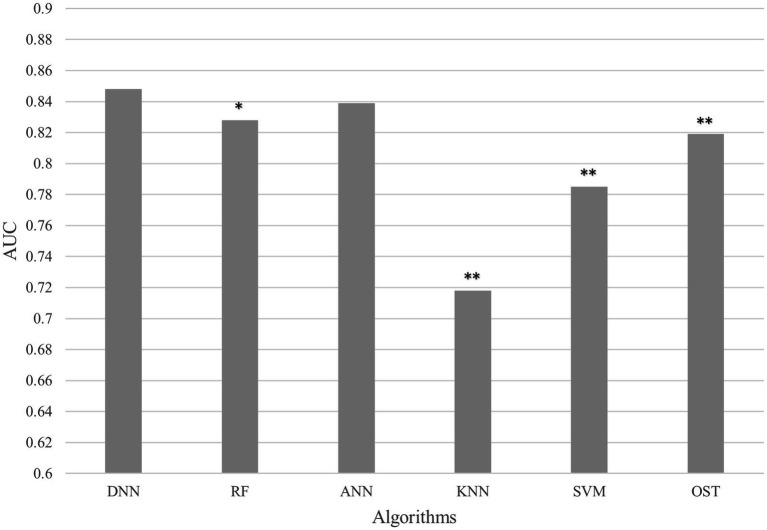
AUC comparison between DNN and other algorithms. Average AUC on 10 hold out test sets of the DNN framework against four ML algorithms (RF, ANN, KNN, and SVM) and the OST model for prediction of osteoporosis risk. The above algorithms were run 10 times on different train/test splits. We used pairwise Wilcoxon signed-rank test to estimate the statistical significance of the difference in performance between DNN and other methods (∗∗ *p* < 0.05, ∗ *p* < 0.1).

To assess the ability of the developed novel DNN framework for predicting osteoporosis, we compared the ROC curves of the developed novel DNN with four commonly used ML models (RF, ANN, KNN, and SVM), as well as the conventional OST model (Figure 1). As shown in Table 3, the developed novel DNN model yielded the best predictive performance (AUC = 0.848) among all the tested methods, with relatively higher sensitivity (0.740) and specificity (0.793). Further statistical analysis demonstrated that the developed novel DNN model has a significant improvement (Wilcoxon signed-rank test *p* < 0.05) in predicting osteoporosis compared to most other tested methods (Figure 2). RF algorithm has the highest predictive accuracy of 0.757, followed by DNN (0.753), ANN (0.747), SVM (0.745), OST (0.731), and KNN (0.651) methods. These findings indicated that the developed novel DNN model was more effective in predicting osteoporosis risk compared to the other methods in this study setting.

**Table 3:** Performance comparison among DNN, OST, and other ML models.

Model	AUC	Accuracy	Sensitivity	Specificity
DNN	0.848	0.753	0.740	0.793
RF	0.828	0.757	0.748	0.785
ANN	0.839	0.747	0.725	0.815
KNN	0.718	0.651	0.642	0.675
SVM	0.785	0.745	0.725	0.808
OST	0.819	0.731	0.714	0.786

### Identification of common important features among the developed novel DNN model and other algorithms

**Figure 3:**
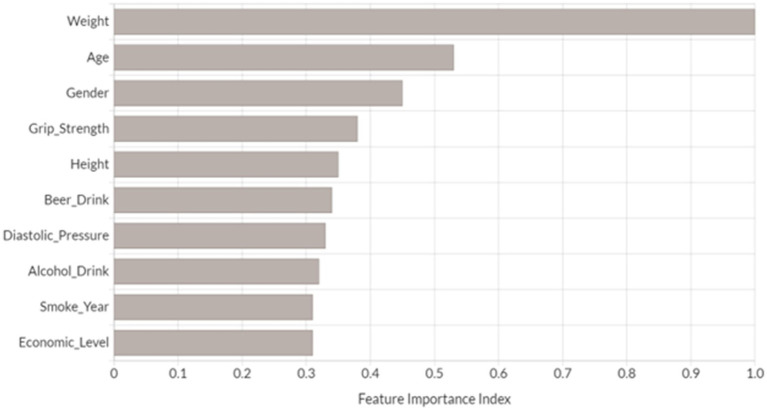
Top 10 most important variables identified by DNN model. Feature importance index was determined by calculating the relative influence of each variable through tree-based model: whether that variable was selected to split on during the tree building process, and how much the squared error (over all trees) improved (decreased) as a result (Candel et al., 2023). Each feature’s importance has been scaled between 0 and 1 based on the most significant feature.

We performed feature importance analysis for the developed novel DNN model. As shown in Figure 3, the top 10 most important features for predicting osteoporosis risk are weight, age, gender, grip strength, height, beer drinking, diastolic pressure, alcohol drinking, smoke years, and economic level. Interestingly, all these features have been either proposed as osteoporosis risk factors or associated with osteoporosis in the previous studies (Grainge et al., 1998; Cappuccio et al., 1999; Henry and Eastell, 2000; Koh et al., 2001; KANTOR et al., 2004; Tucker et al., 2009; Kim et al., 2012; du et al., 2017). Furthermore, nine out of the top 10 features (except for alcohol drinking) from the developed novel DNN model were also identified in the top 10 features from one or more of the other tested ML models (Supplementary Figures 3A–D). Among them, five risk factors were presented in the top 10 ranked features across all ML models, including weight, age, grip strength, height, and smoke years. Relative to the subjects in the non-osteoporosis risk group, subjects with osteoporosis risk were of significantly higher age and smoke years, and lower weight, height, and grip strength (Wilcoxon signed-rank test *p* < 0.05).

### The optimal number of sample size and important variables for osteoporosis risk prediction

**Figure 4:**
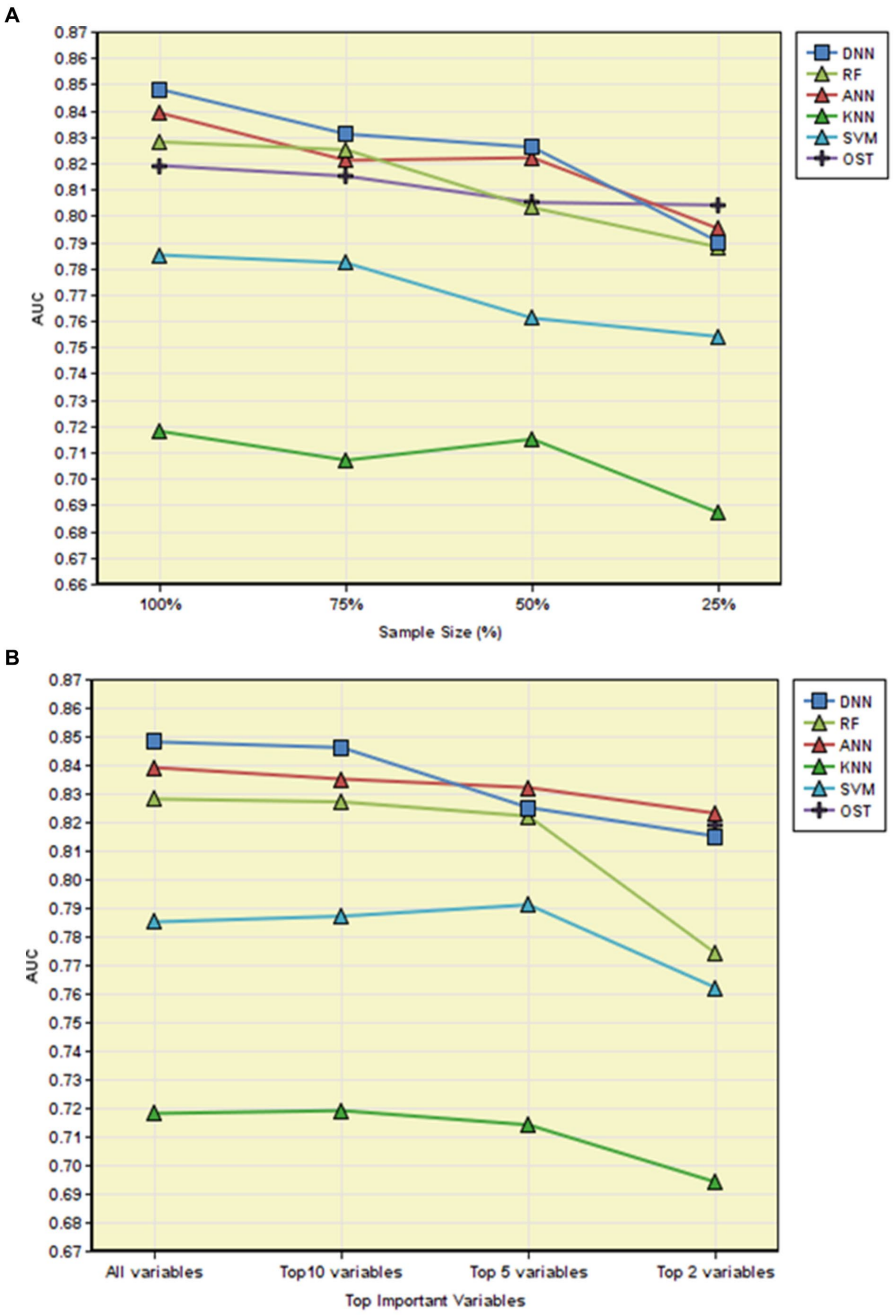
AUC comparison among different models. **(A)** AUC comparison among different models with different sample sizes; **(B)** AUC comparison among different models with different numbers of variables. OST applies a simple regression model based on age and body weight.

To assess the effects of sample size on the predictive performance of the tested models, we randomly subsampled 75, 50, and 25% of the original data set, corresponding to approximately 6,000, 4,000, and 2,000 subjects, respectively. As expected, decreasing in sample size generally led to reduced AUCs in all classification methods (Figure 4A). Notably, the developed novel DNN and ANN models still performed fairly well (AUC _DNN_ = 0.826, AUC _ANN_ = 0.826) when sample size was reduced to 50% of the original data set, but the performances were dropped dramatically when the sample size was decreased from 50 to 25% of the original data set (Wilcoxon signed-rank test *p* < 0.05). Interestingly, OST achieved the best AUC (0.804) among all the tested models when the sample size is around 25% of the original samples (*n* = 2,000).

In parallel, we investigated the predictive performances of the tested models when only utilize the top 10, 5, and 2 most important features identified by each corresponding algorithm. As shown in Figure 4B, we observed that even with reduced numbers of features, the developed novel DNN model and the robust ML methods (ANN and RF) still had fairly good performances, especially when using the top 10 most important variables for prediction. For example, the simplified novel DNN model with top 10 risk factors obtained a comparable discriminatory power (AUC _top10 variables_ = 0.846) against the original novel DNN model (AUC _all variables_ = 0.848).

## Discussion

Making accurate predictions of disease risk can be of great clinical value for healthcare professionals. A highly effective data-driven predictive algorithm is desired to increase the efficiency of disease prevention and improve patient outcomes through early detection and treatment. In the past decade, a wide range of conventional statistical models and ML algorithms have been developed for osteoporosis risk assessment. However, these models/algorithms do not adequately fulfill the need of clinicians to accurately predict osteoporosis risk. Recently proposed DNN approach, which increases the depth of the neural network and adds parameters to make more adjustments to the input variables, has achieved record-breaking performance in a variety of clinical implementations, such as medical imaging analysis, and disease prediction/classification. Nevertheless, little progress has been achieved so far with respect to the development of DNN model for osteoporosis risk prediction. To fill this gap, we developed and constructed a novel DNN framework for osteoporosis risk prediction, and compared its performance with several previously developed risk prediction models, including four widely used ML techniques and the conventional tool OST. We also tested the feature importance for the developed novel DNN algorithm and identified the common important features among this novel DNN and other algorithms. At last, we assessed the optimal number of important features and sample size for osteoporosis risk prediction. To the best of our knowledge, this is the first report on application of DNN for osteoporosis risk assessment in the aging population with large sample size.

By using extensive demographic and routine clinical data of 8,134 subjects with age more than 40 from the LOS, we demonstrated that the developed novel DNN model has the best performance (AUC = 0.848) among all the tested models in distinguishing osteoporosis risk subjects. The top 10 most important features of osteoporosis identified by the developed novel DNN include weight, age, gender, grip strength, height, beer drinking, diastolic pressure, alcohol drinking, smoke years, and economic level. Consistent with previous reports (Koh et al., 2001), our results also suggested that low body weight and older age are the most important risk factors for the development of osteoporosis. Although many clinical studies have shown that low grip strength correlates with low BMD and higher risk of fragility fractures (Kim et al., 2012), there has been no study using grip strength as a predictor for osteoporosis risk prediction. Interestingly, in the current study, grip strength ranked in the top 10 risk factors across all the ML models. The effect of grip strength on BMD may be explained by the mechanostat theory since the bones adapt not only to static forces (of excessive weight), but also to the dynamic forces created by muscular contractions (Frost, 2003). Previous studies have suggested close interactions between skeletal muscle and bone metabolism (Kaji, 2013). For example, muscle-derived local growth factors, IGF-I and IGF-binding protein-5, may affect bone metabolism in an anabolic fashion and ultimately enhance bone formation (Kaji, 2013). We also identified two discriminative variables, alcohol and beer drinking, in the top 10 most important features for osteoporosis. Interestingly, moderate alcohol consumption can decrease the turnover of bone for women, which may benefit their bone health and lower the risk of developing osteoporosis (Sampson, 2002). Also, previous study showed that dietary intake of silicon, a major constituent of beer (Sripanyakorn et al., 2004), was positively associated with BMD in osteoporotic women (Eisinger and Clairet, 1993; Jugdaohsingh et al., 2002), and this has since been confirmed in a sample of men and premenopausal women in the Framingham study (Jugdaohsingh et al., 2004). Nevertheless, in the current study, we only considered the dichotomous status (yes/no) of current alcohol or beer drinking due to the limited questionnaire information. Therefore, the future tasks of model building should attempt to distinguish the effect of varying levels of alcohol/beer consumption on osteoporosis risk prediction.

Interestingly, a recent study of osteoporosis risk prediction using a dataset of 10,000 patient records, including chronic condition/disease, identified several influential variables, such as age, gender, lipid disorders, cancer, and Chronic Obstructive Pulmonary Disease (COPD) (Tu et al., 2024). Indeed, their findings align with our results that both age and gender are significant risk factor of osteoporosis. Notably, lipid disorders, such as high low-density lipoprotein (LDL) cholesterol and triglycerides, are linked to increased risk of cardiovascular diseases including hypertension. Elevated lipids can lead to arterial plaque formation and stiffness, contributing to higher diastolic blood pressure. This relationship underscores the importance of managing lipid levels to reduce cardiovascular risks, including hypertension-related complications (Zhang et al., 2019; Baba et al., 2023). Furthermore, the well-established relationship between smoking duration (measured in pack-years) and COPD highlights the substantial impact of lifelong smoking on respiratory health. Studies indicate that lifelong smokers have a 50% probability of developing COPD during their lifetime, with early smoking initiation exacerbating adverse clinical outcomes such as compromised lung function, increased mortality risk, and cardiac changes like thicker left ventricular diameters (Laniado-Laborin, 2009). Effective smoking cessation strategies are paramount, yet their efficacy varies among COPD patients based on their smoking history (Laniado-Laborin, 2009). Despite these challenges, both studies demonstrate the effectiveness of machine learning models in early detection and risk stratification for osteoporosis, offering promise for personalized prevention and management approaches in clinical practice.

For any predictive model to be practical in making clinical decision, it should avoid model complexity and use data that can be easily assessed by clinicians at the time of triage, it may not be necessary to add all variables even though they perhaps have some influences on predictive performance. Therefore, to optimize the risk factors and simplify the predictive model, we tested the effects of varying number of important features on performance of osteoporosis prediction. Notably, by using the top 10 most important features instead of all the risk factors, we observed only a negligible reduction of the predictive performance in the developed novel DNN model. This suggested that our simplified novel DNN model may be an optimal choice for predicting osteoporosis risk in clinical practice. In addition, we tested the effects of sample size on model performance and found that the developed novel DNN model still performed well when sample size was approximately 4,000. Interestingly, OST achieved the best performance (0.804) among all the tested models when sample size decreased to approximately 2,000, that there is no universal best predictive model across all conditions and the OST may be a preferred model when only very limited features are available, and the sample size is relatively small. Moreover, we explored the predictive performance of the developed novel DNN model with both reduced sample size and limited input features, and found that our DNN model can still achieve an AUC of 0.821 when utilizing the top 10 most important variables with approximately 4,000 subjects, which was better than or comparable to the performances of most other ML algorithms using the full dataset.

Comparing the results from the previous studies on osteoporosis risk prediction using various machine learning models provides a broad view of the current capabilities and advancements in this field. Sadatsafavi et al. (2005) focused on using ANN and linear regression to predict BMD in Iranian post-menopausal women. They found that as the number of input variables increased, ANN outperformed linear regression models, especially for larger datasets (2,158 participants), demonstrating better accuracy and predictive power as measured by AUC. Eller-Vainicher et al. (2011) assessed the effectiveness of ANN versus logistic regression in diagnosing severe degrees of osteoporosis among 372 postmenopausal women, ANN demonstrated superior prognostic performance utilizing 45 clinical variables. Specifically, in distinguishing women with any degree of osteoporosis from those without, ANNs achieved a sensitivity of 72.5% and accuracy of 75.5%. Xu et al. (2013) proposed a novel method using micro-CT images analyzed through SVM and KNN, achieving excellent diagnostic precision, recall, and F-measure. This approach emphasized the effectiveness of combining multiple image-derived features for highly accurate classification between osteoporotic and normal cases. In our study, we assessed the performance of a DNN in predicting osteoporosis risk using comprehensive data from over 8,000 participants. The DNN model outperformed other machine learning models (RF, ANN, KNN, SVM) and a traditional regression model in predicting BMD T-scores, achieving the highest AUC (0.848). Importantly, the DNN model maintained high predictive performance even with reduced sample sizes and fewer variables, highlighting its efficiency and robustness. In summary, while all studies demonstrate the growing effectiveness of machine learning models in osteoporosis prediction and diagnosis, the DNN model in our study showed exceptional adaptability and high performance across different conditions. These results suggest that deep learning could be particularly beneficial in clinical settings for early diagnosis and intervention of osteoporosis, leveraging complex and extensive datasets to improve prediction outcomes.

The predictive models for osteoporosis developed in this study has significant implications for global public health and primary healthcare. Firstly, the use of predictive models such as the DNN can greatly impact global public health by enabling early diagnosis and intervention for osteoporosis. Early detection of osteoporosis risk allows for timely preventive measures and interventions, which can help mitigate the progression of the disease and reduce associated complications such as fractures. This, in turn, can contribute to improving overall health outcomes and quality of life for individuals at risk of osteoporosis. Moreover, the application of advanced predictive models like the DNN in primary healthcare settings holds promise for enhancing healthcare delivery. Primary healthcare providers can leverage such models to assess osteoporosis risk in general population more accurately, leading to personalized screening and intervention strategies for their patients. This can lead to more efficient use of healthcare resources, improved patient outcomes, and reduced healthcare costs associated with osteoporosis-related complications. Overall, this study not only advances our understanding of osteoporosis risk prediction but also has the potential to make a significant impact on public health by offering effective tools and strategies for early diagnosis and intervention of osteoporosis and/or osteoporosis-associated fracture, particularly in primary healthcare settings.

One of the main limitations of our study is that we only focus on demographic and routine clinical data assessed by questionnaires. To further enhance the predictive accuracy and interpretability of osteoporosis risk prediction models, future studies can attempt to construct a comprehensive prediction model by including additional factors, such as multivariate time series variables, blood biochemical markers (e.g., fasting blood glucose and serum lipid levels etc.), as well as the simultaneous prediction of osteopenia and osteoporosis using multi-category classification. On the other hand, we should also consider model complexity and feasibility and difficulty in acquiring these data in clinical practice. Furthermore, our study was carried out at a single institution. Although the sample size is the largest so far in the bone field, further external validation work with other independent populations should be designed to assess the performance and generalizability of our proposed novel DNN model. Lastly, although we implemented several precautions in our study design and analysis, such as meticulously choosing a diverse dataset from the LOS to encompass various demographic and clinical variables, we recognize that the impact of traditional clinical protocols on data balance and biases remains a hurdle in predictive modeling. Future investigations may delve into strategies to tackle these limitations more comprehensively, such as integrating supplementary data sources or crafting robust preprocessing techniques to manage biased data more adeptly.

## Conclusion

In conclusion, the novel DNN model that we developed and constructed in this study has a good performance in osteoporosis risk prediction compared with other ML models. It may serve as a cost-effective prescreening tool to determine candidates for evaluation with DXA and help clinicians to initiate early preventive actions for osteoporosis-related fracture.

R Packages and Source Code

- *mice* (https://github.com/amices/mice)
- *corrplot* (https://cran.r-project.org/web/packages/corrplot/vignettes/corrplot-intro.html)
- *h2o* (https://docs.h2o.ai/h2o/latest-stable/h2o-docs/faq/r.html)
- *caret* (https://topepo.github.io/caret/)

## Data availability statement

The raw data supporting the conclusions of this article will be made available by the authors, without undue reservation.

## Ethics statement

The studies involving humans were approved by Tulane University the Ethics Review Board. The studies were conducted in accordance with the local legislation and institutional requirements. The participants provided their written informed consent to participate in this study.

## Author contributions

CQ: Conceptualization, Data curation, Formal analysis, Investigation, Methodology, Project administration, Software, Supervision, Validation, Visualization, Writing – original draft, Writing – review & editing. KS: Data curation, Investigation, Writing – original draft. ZL: Data curation, Investigation, Writing – original draft. QT: Data curation, Resources, Validation, Writing – original draft. LZ: Resources, Writing – original draft. LW: Resources, Writing – original draft. HD: Funding acquisition, Project administration, Writing – review & editing. HS: Funding acquisition, Project administration, Supervision, Writing – review & editing.

## Footnotes

^1^ https://cran.r-project.org/web/packages/mice/

^2^ https://cran.r-project.org/web/packages/corrplot/

^3^ https://cran.r-project.org/web/packages/caret/
