# Supplementary material for: Developing and comparing deep learning and machine learning algorithms for osteoporosis risk prediction
Source: Front Artif Intell. 2024 Jun 11;7:1355287. doi: 10.3389/frai.2024.1355287 (PMC11196804; doi:10.3389/frai.2024.1355287)
Supplement: Supplementary file 1 [file Data_Sheet_1.docx]

**Supplementary Materials**

**Comparing Deep Learning and Machine Learning Algorithms for**

**Osteoporosis Risk Prediction**

**Contents**

**Sample Description**

All the subjects used in this study were recruited through the Louisiana Osteoporosis Study (LOS), a repertoire of more than 17,000 subjects (by end of October 2023) collected for investigating genetic and environment risk and protective factors for osteoporosis in Southern Louisiana. Subjects age 18 and over were recruited in New Orleans, Baton Rouge, and surrounding areas in Louisiana, USA. A set of exclusion criteria was applied for the LOS recruitment (all the ~17,000 subjects) to exclude subjects with known disease/conditions that may affect bone metabolism. The detailed inclusion and exclusion criteria are listed in the following. All the conditions were assessed with questionaire that are based on known medical history of subjects.

# *Subject Inclusion Criteria*

(1) be at least 18 years of age; (2) be willing to participate in the study, have a bone densitometry exam, and blood drawn; and (3) speak and understand spoken English.

# *Subject Exclusion Criteria*

(1) female subjects who are, or could be pregnant; (2) female subjects who have had bilateral oophorectomy; (3) serious residuals from cerebral vascular disease; (4) diabetes mellitus, except for those controlled under medication; (5) chronic renal failure; (6) chronic liver failure; (7) significant chronic lung disease; (8) alcohol abuse as defined by those who cannot limit drinking, regularly become intoxicated, and cannot fulfill major responsibilities at work, school, or home; (9) chronic obstructive pulmonary disease (COPD); (10) corticosteroid therapy at pharmacologic levels for more than 6 months duration; (11) treatment with anticonvulsant therapy for more than 6 months duration; (12) evidence of other metabolic or inherited bone disease such as hyper- or hypoparathyroidism, Paget’s disease, osteomalacia, osteogenisis imperfecta or others; (13) rheumatoid arthritis (except for minor cases that involve only hand joint and wrist); (14) collagen diseases (i.e., Osteogenesis imperfecta and Hypochondrogenesis); and (15) chronic gastrointestinal diseases including celiac disease, postgastrectomy, Crohn’s disease, ulcerative colitis, liver transplant, and cirrhosis.

**Sample Measurements and Labeling**

Height was measured in a standing position using a calibrated Health-O-Meter Professional height and weight scale without shoes. Weight was measured with a balance beam, calibrated Health-O-Meter Professional height and weight scale. Body mass index (BMI) was calculated by dividing weight (kg) by height squared (m^2^).

Questionnaires were used to collect data including age, gender, race/ethnicity, socioeconomic status, and behavioral factors. Self-reported race/ethnicity was identified by selection from the following groups: Caucasian/White, African-American/Black, Asian, and Hispanic/Latino. Socioeconomic status was measured by education level and personal annual income; specifically, for education participants could select from: eighth grade or less, some high school, high school graduate, some college, college graduate, and graduate degree. We condensed education into the following four categories: less than high school graduate, high school graduate, college (including some college and college graduate), and graduate level. Participants selected personal annual income from five scales—under $20,000, $20,000–39,999, $40,000–59,999, $60,000−79,999, and $80,000 or more. The five income categories were referred to as Levels 1, 2, 3, 4, and 5, respectively with higher levels indicating higher income.

Behavioral factors included regular exercise, smoking, milk consumption, alcohol use, and hormone replacement therapy etc. Regular exercise was measured by responding to, “Do you currently exercise on a fairly regular basis?” “Do/did you smoke cigarettes? — Past history or current use of tobacco should be YES” was used to assess smoking status. Milk consumption was assessed by the question, “Do you drink milk (including fortified soy, rice, almond milks, etc.)?” “Do you have a history of alcoholism or a drinking problem?” was used to assess alcohol use. A response of yes to the question was considered as having a drinking problem. Menopausal hormone therapy was measured by asking, “Do you routinely accept hormone replacement therapy?” Although questions, such as duration and intensity, were asked for behavioral factors, the responses rate was relatively low. Therefore, behavioral factors were expressed as “yes/no”.


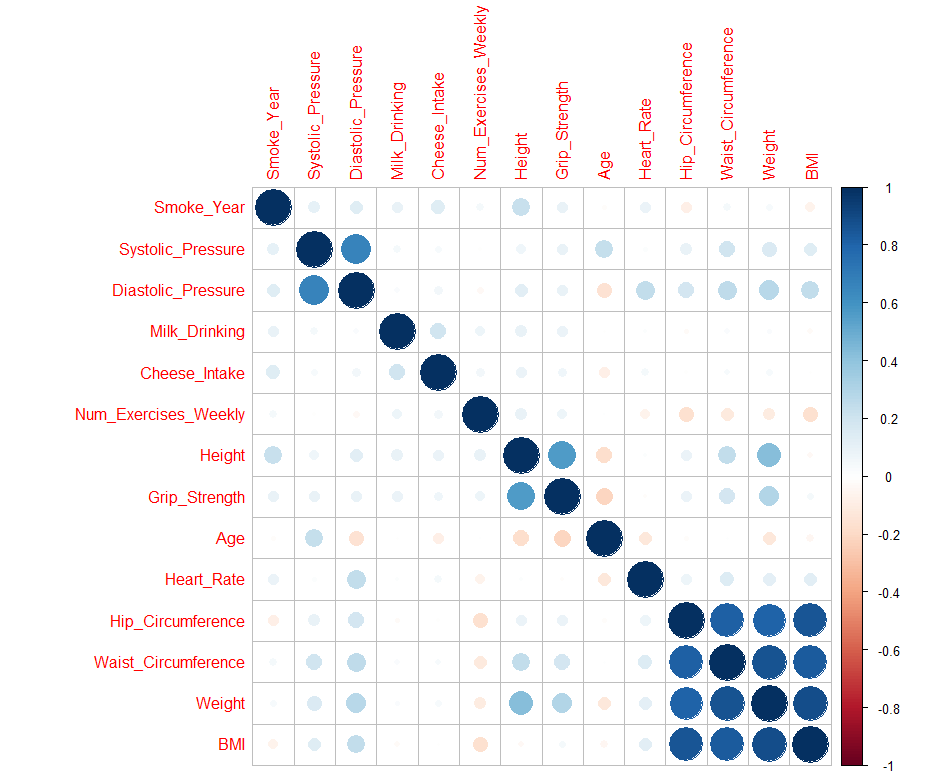

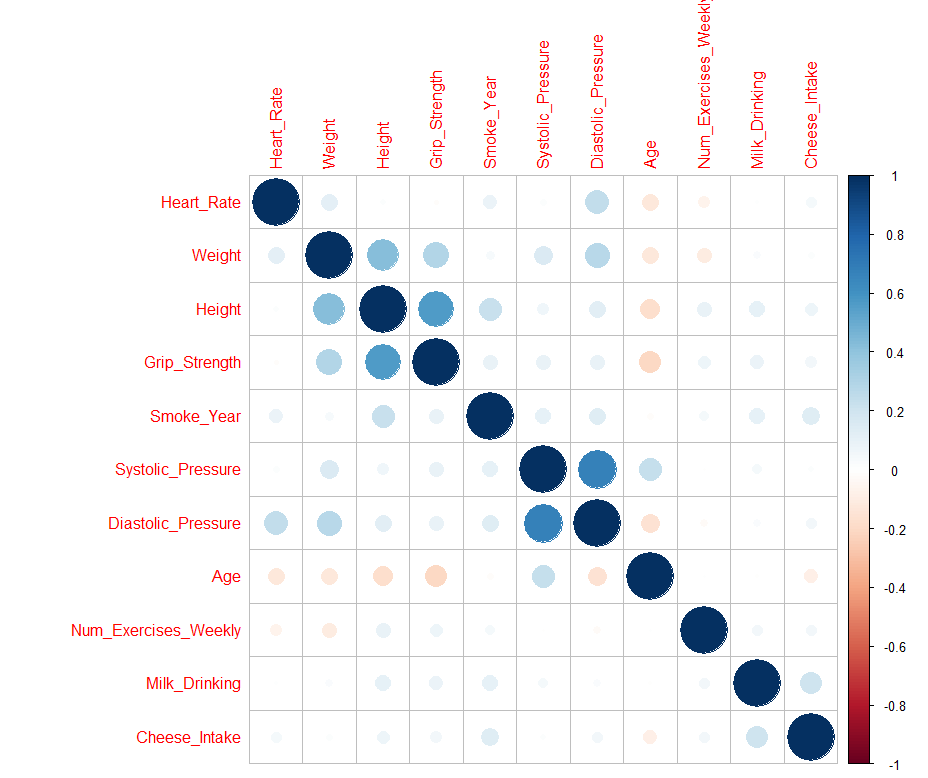


**Supplementary Figure 1. Feature selection analysis for continuous variables.** The correlations between continuous variables were calculated and visualized with the R package *corrplot*. Three variables (BMI, waist circumference, and hip circumference) which were highly correlated (Pearson’s correlations > 0.70) with weight were removed to smooth out the noise and simplify the model. A total of 11 continuous variables were used in the following analysis.

**Supplementary Figure 2A**

**
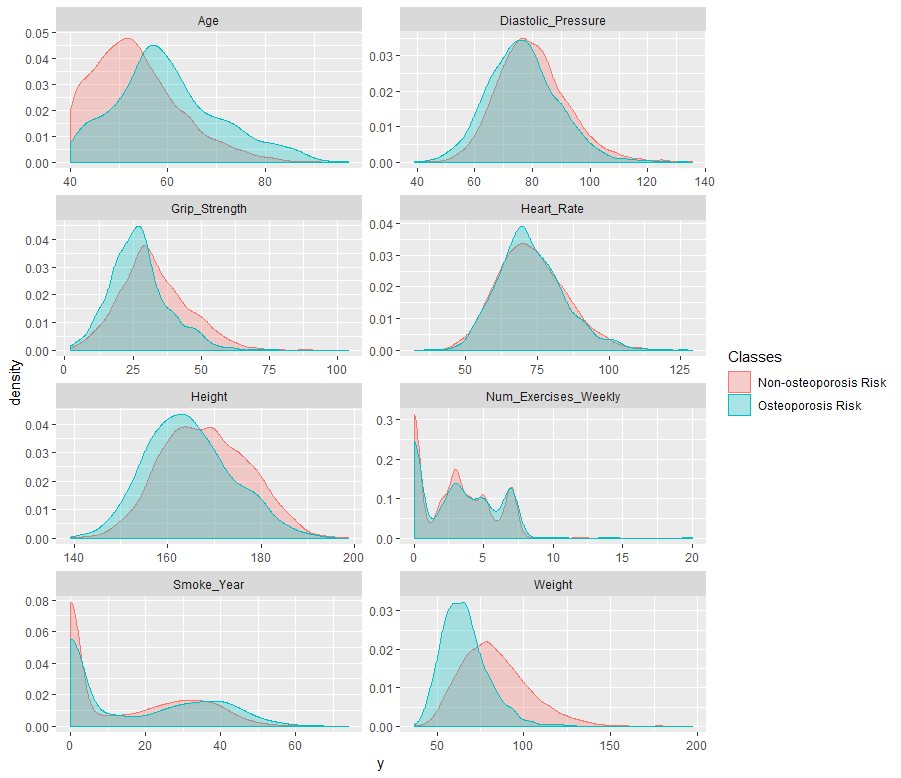
**

**Supplementary Figure 2B
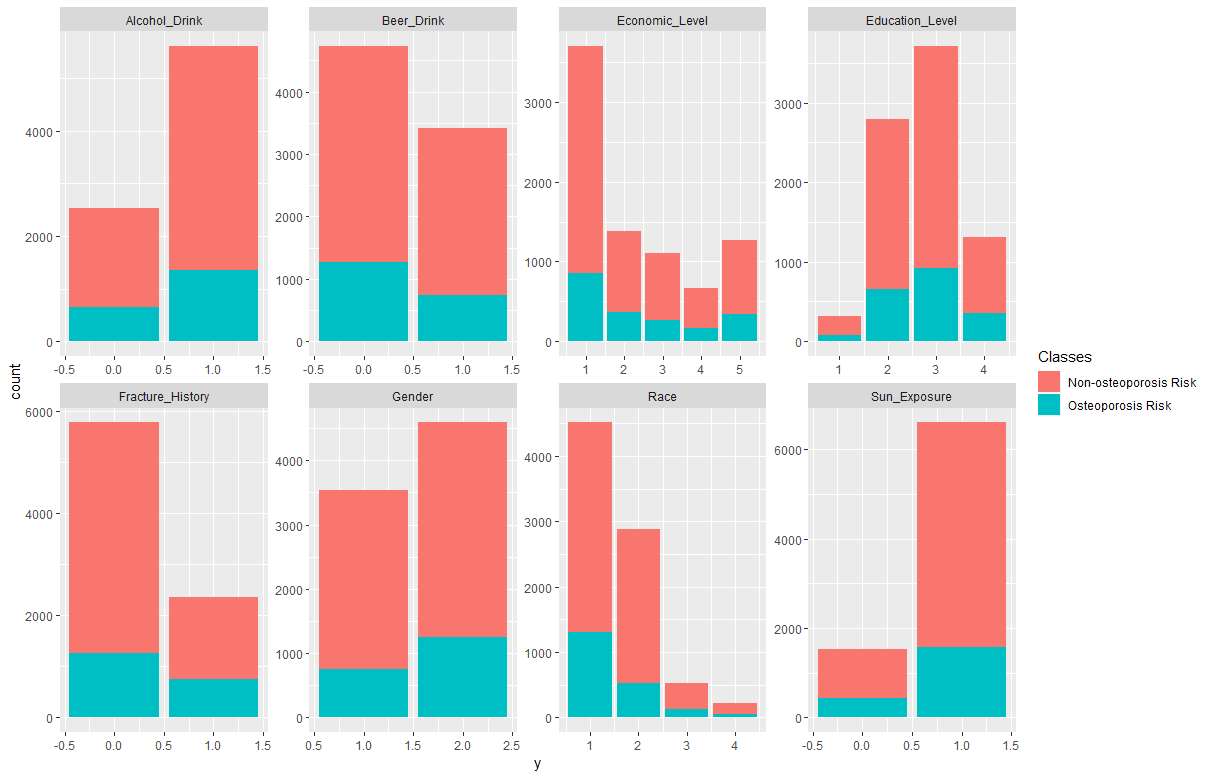
**

**Supplementary Figure 2. Distribution for continuous variables (A) and categorical variables (B)**

**Supplementary Figure 3A**

**
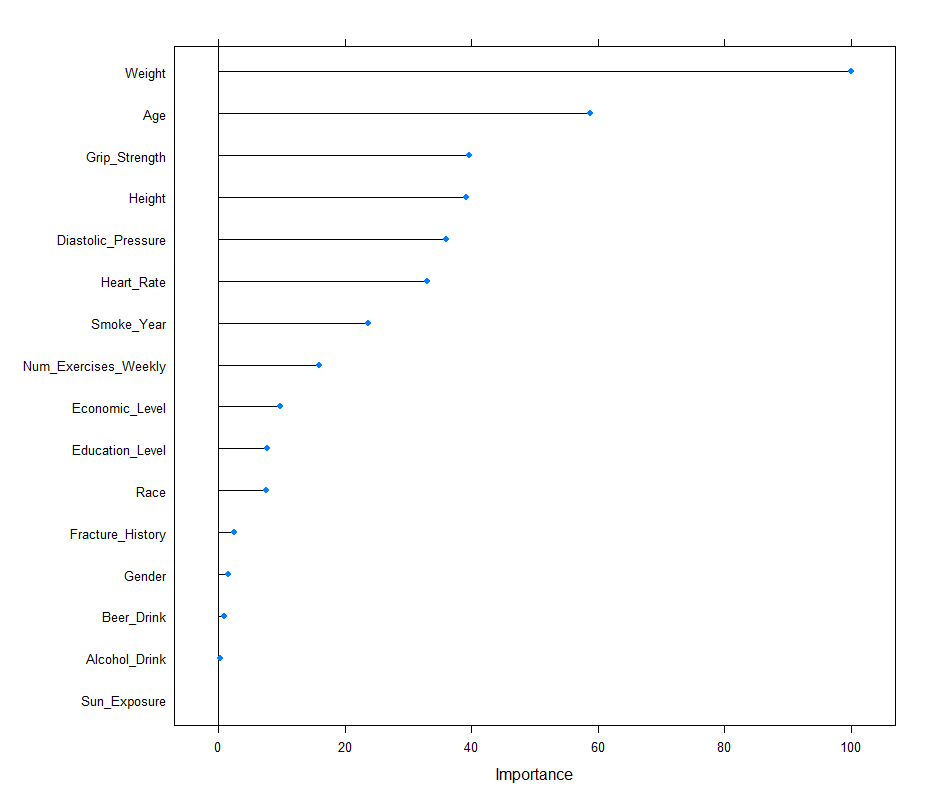
**

**Supplementary Figure 3B**

**
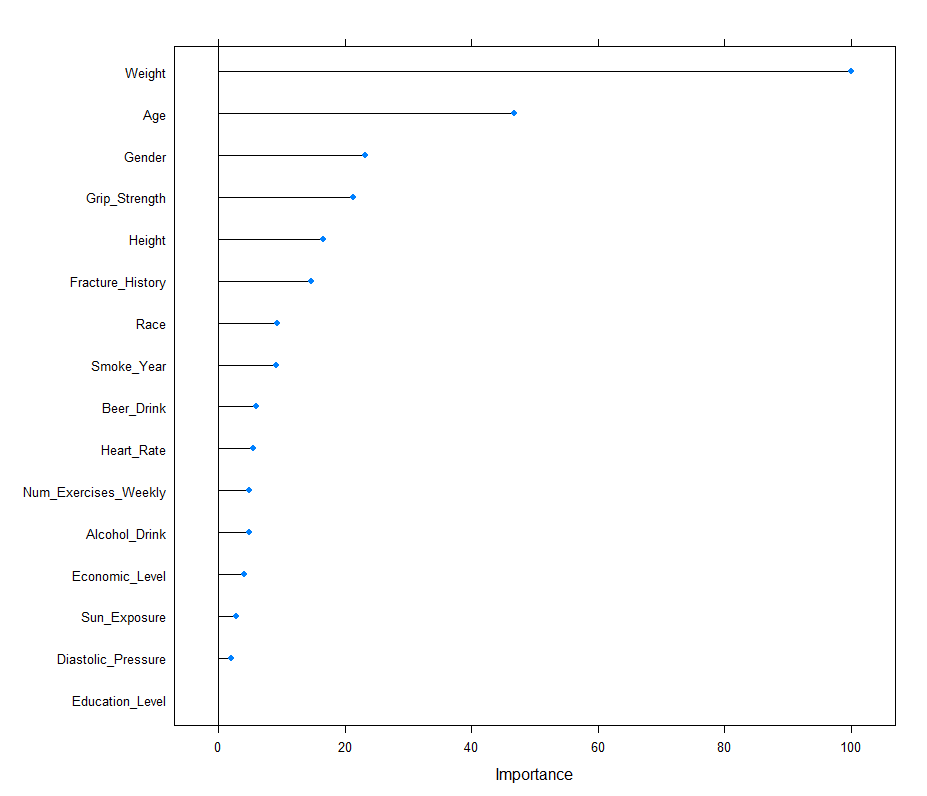
**

**Supplementary Figure 3C**

**
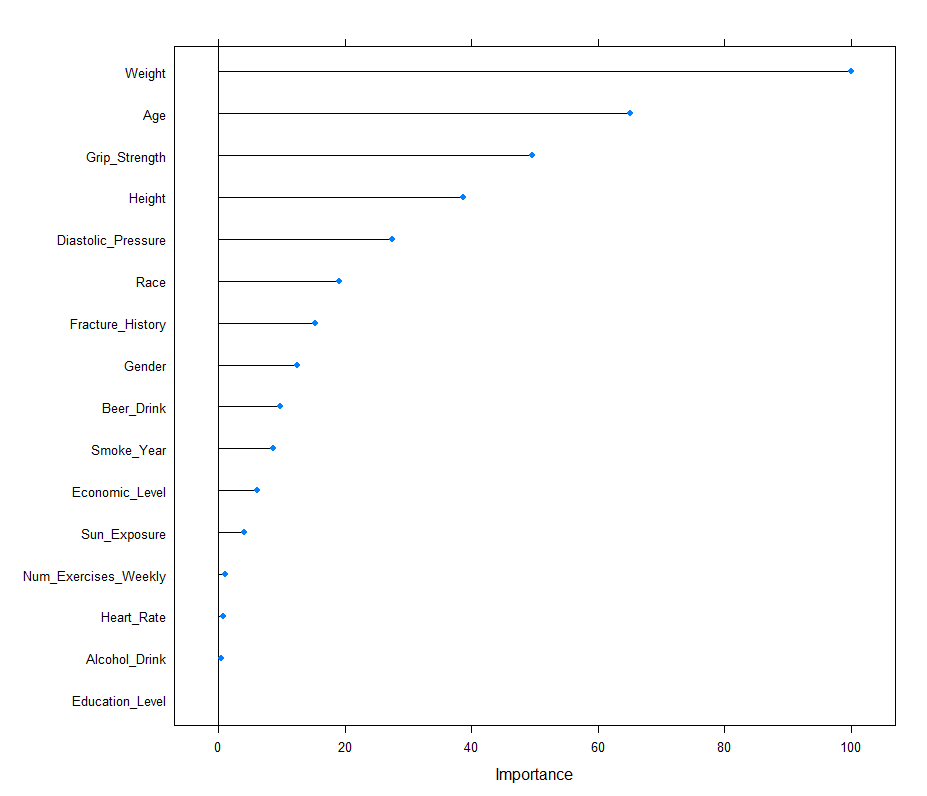
**

**Supplementary Figure 3D**

**
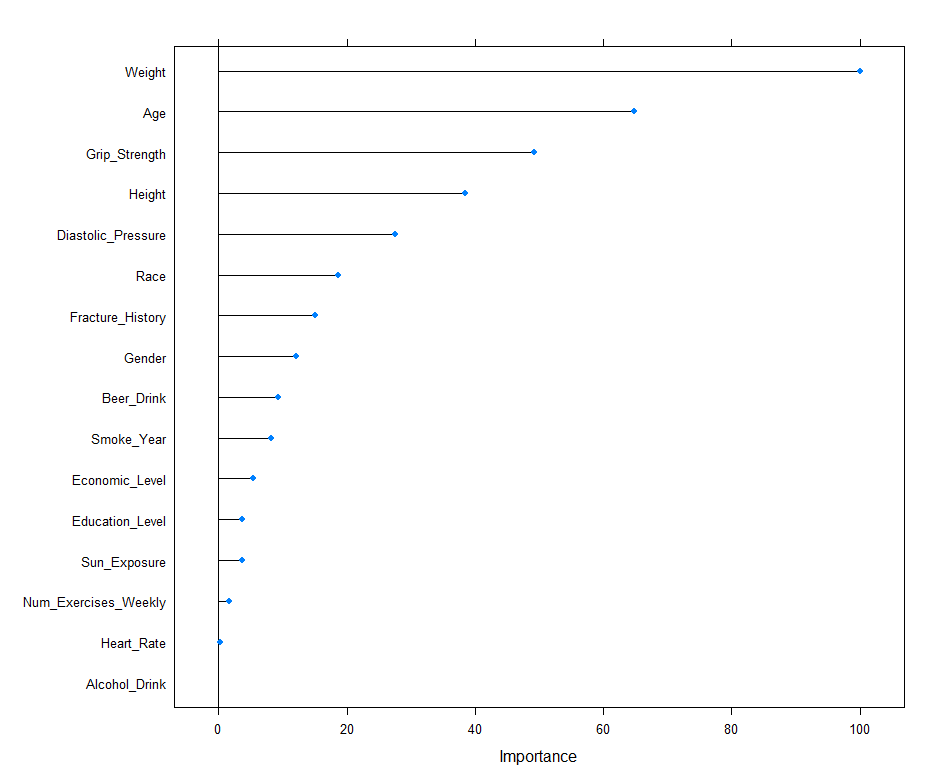
**

**Supplementary Figure 3. Feature importance for RF (A), ANN (B), DNN (C), and SVM (D)**
